# The implication of *dihydrofolate reductase *and *dihydropteroate synthetase *gene mutations in modification of *Plasmodium falciparum *characteristics

**DOI:** 10.1186/1475-2875-6-108

**Published:** 2007-08-08

**Authors:** Ishraga E A-Elbasit, Michael Alifrangis, Insaf F Khalil, Ib C Bygbjerg, Emad M Masuadi, Mustafa I Elbashir, Hayder A Giha

**Affiliations:** 1 Malaria Research Centre (MalRC), Department of Biochemistry, Faculty of Medicine, University of Khartoum, PO Box 102, Khartoum, Sudan; 2 Centre for Medical Parasitology (CMP) at Institute of Medical Microbiology and Immunology and Institute of Public Health, University of Copenhagen, Denmark; 3 Department of Biochemistry, and Department of Family & Community Medicine, Faculty of Medicine and Medical Sciences, Arabian Gulf University (AGU), PO Box 26671, Manama, Kingdom of Bahrain

## Abstract

**Background:**

The *Plasmodium falciparum *dihydrofolate reductase (DHFR) and dihydropteroate synthetase (DHPS) are enzymes of central importance in parasite metabolism. The *dhfr *and *dhps *gene mutations are known to be associated with sulphadoxine/pyrimethamine (SP) resistance.

**Objective:**

To investigate the effects of *dhfr/dhps *mutations on parasite characteristics other than SP resistance.

**Method:**

Parasite infections obtained from 153 Sudanese patients with uncomplicated falciparum malaria treated with SP or SP + chloroquine, were successfully genotyped at nine codons in the *dhfr/dhps *genes by PCR-ELISA.

**Results & conclusion:**

Mutations were detected in *dhfr *at N51I, S108N and C59R, and in at *dhps *at A/S436F, A437G, K540E and A581G, the maximum number of mutations per infection were five. Based on number of mutant codons per infection (multiplicity of mutation, MOM), the infections were organized into six grades: wild-types (grade 0; frequency, 0.03) and infections with MOM grades of 1 to 5, with the following cumulative frequency; 0.97, 0.931, 0.866, 0.719, 0.121, respectively. There was no significant association between the MOM and SP response. Importantly, immunity, using age as a surrogate marker, contributed significantly to the clearance of parasites with multiple *dhfr/dhps *mutations. However, these mutations have a survival advantage as they were associated with increased gametocytogenesis. The above implications of *dhfr/dhps *mutations were associated with MOM 2 to 5, regardless of the gene/codon locus.

## Background

Falciparum malaria is still out of control, primarily because of the ability of the parasite to develop resistance against the used drugs. But, also the fast disseminations of the resistant parasites and possibly the accelerated ability of the parasite to develop resistance against new drugs, are important factors [1]. Sulphadoxine/pyrimethamine (SP), has been an alternative to CQ for treatment and control of uncomplicated malaria in endemic countries, it was effective, affordable and complying drug. The fixed combination in SP inhibits the action of two enzymes, dihydrofolate reductase (DHFR) and dihydropteroate synthetase (DHPS) in folate metabolism pathway [2,3]. Mutations in the parasite genes coding for the two enzymes, *dhfr *and *dhps*, lead to SP resistance, however, the mutations also affect the parasite population, fitness and evolution [4,5].

Although elimination of the asexual stages of *Plasmodium falciparum *is the focus of the treatment of individual symptomatic patients, at population level, reducing the carriage of gametocytes is necessary to limit the transmission of malaria parasites and the spread of anti-malarial drug resistance. In relation to SP treatment, the gametocyte carriage and infectivity to mosquitoes was found to be consistently higher in patients infected with drug resistant compared with drug sensitive malaria parasites [6].

In malaria endemic areas, the host immune system acts synergistically with chemotherapy in the clearance of parasites [7]. The susceptibility of parasites with mutations to the host immune mechanisms is largely unknown. In studies done in Mali, the term genotype-resistance index (GRI) was introduced to correct for the differences between the two arms of the equation; the treatment failure rate and the prevalence of parasite mutations [8]. This index is a proxy for the role of immunity, which varies between epidemiological settings, and in the same setting depends on the age. The hypothesis on which this study was based was a combination of two old observations, the association of drug resistance with *dhfr/dhps *mutation, and the implication of immunity in clearance of drug resistant parasites. That implies, the *dhfr/dhps *mutant parasites might be more susceptible than the wild variants to host immune clearance. Further more the study aim to investigate the relationship between the *dhfr/dhps *mutations and the parasite fitness in term of; *in-vivo *parasite growth and gametocytogenesis. This data is complementary to a previous report in which these parameters were analysed in relation to *in-vivo *drug response [9].

## Methods

### Study area, population and parasites

The study was carried out in Daraweesh and Kajara villages in Eastern Sudan, the details is mentioned elsewhere [9]. The major characteristics of this setting are seasonality and marked instability of malaria transmission, and susceptibility of people in all age groups to malaria infection. This study was conducted in 2003, one year before the official replacement of CQ with artesunate/SP combination therapy as first-line treatment for malaria.

All patients with uncomplicated *P. falciparum *malaria (n, 254) in all age groups were enrolled in the study, excluding pregnant women and severely ill patients. A modified protocol for assessment of drug resistance was used [10]. The study design, *in-vivo *results and classification of treatment outcome into: early treatment failure (ETF), late treatment failure (LTF), and adequate clinical and parasitological response (ACR), were presented before [9]. However, PCR was not done to confirm that all microscopically confirmed ACPR infections were not having sub-microscopic parasitaemia. Blood collected in filter papers during 28-day follow up was used for genotyping to confirm recrudescence.

A subset of 168 infections, obtained from patients with (TF, 82) and randomly selected patients with (ACR, 86), were genotyped in the *P. falciparum *genes *dhfr *and *dhps*. Only 153 parasite infections were successfully genotyped at all examined loci, 78 from patients with ACR and 75 from patients with TF (included 17 ETF, and the remaining were LTF). The informed consent of the patient or guardian was obtained, and institutional and national ethical clearance were obtained from the Sudan Ministry of Health.

### DNA extraction, PCR and SSOP-ELISA for detection of drug-resistance-associated mutations

The parasite DNA was prepared from filter papers as described before [11]. Single nucleotide polymorphisms (SNP) and haplotypes of the SP resistance genes; *dhfr *and *dhps *(SP), were analysed by a recently developed PCR-SSOP-ELISA method [12]. The laboratory clones; 3D7, 7G8, Dd2, FCR3 and K1, and four field infections expressing the 164L of *dhfr *and 436/437AA, 436/437AG and 540E genotypes of *dhps*, respectively, were used as a standard positive control. Known SNPs of *dhfr *and *dhps *are represented in this panel of parasites. The nested PCR followed by SSOP-ELISA were performed as described before [12].

### The multiplicity of mutations (MOM)

**Figure 1:**
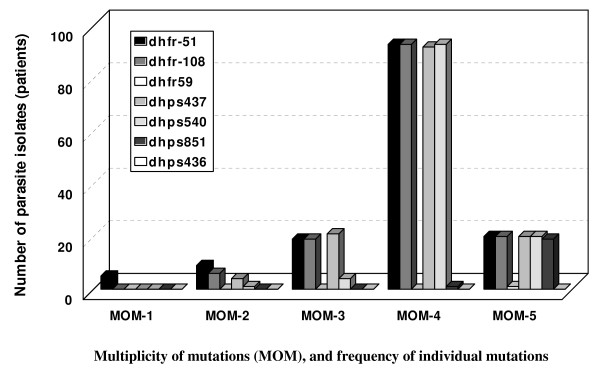
The distribution of the individual *dhfr/dhps *mutant codons (small bars with different shading grade) in parasites infections with different MOM (multiplicity of mutations) grades (multi-bar columns) was shown numerically in y-axis. The parasite infections were obtained from patients with uncomplicated malaria (n = 153), genotyped at *dhfr/dhps *loci, and grouped into five groups based on the number of mutations per parasite (MOM 1 – 5), as indicated by in x-axis. The type of mutant codons were shown in the key.

Based on the number of *dhfr/dhps *mutations regardless of their loci (multiplicity of mutations – MOM), parasite infections were grouped into six grades, wild infections (MOM = 0), and infections with 1, 2, 3, 4 or 5 mutations (MOM 1 to 5). Some parasite infections were negative for one or more of the examined *dhfr/dhps *loci (n = 15) and they were excluded from MOM grouping. The types of mutations in the different MOM grades are shown in Figure 1. In Table 1 a cumulative MOM classification was used to calculate the GRI and to estimate the prevalence of mutations in relation to the treatment outcome. Parasites of a cumulative MOM grade of 1, were parasites had at least one *dhfr/dhps *mutation, by definition this includes all parasites with any number of mutation (MOM 1–5), and parasites of cumulative MOM 2 includes parasites with MOM 2–5 and etc.

**Table 1:** The frequency of the different *dhfr/dhps *multiple mutations (multiplicity of mutations, MOM), in parasite infections obtained from patients with different treatment outcome, ACR and TF, following SP and CQ therapy, and the overall prevalence rate of MOM and the corresponding mutation/TF ratio.

Multiplicity of mutation (MOM)	Frequency of cumulative MOM			Overall prevalence of cumulative MOM		Mutation/TF ratio (GRI)
					
	ACR (n,78)	TF (n,75)	P-value Chi-Square	measured N_1 _= 153	calculated N_2 _= 231	
Null (wild)	0.038 (3)	0.013 (1)	0.640	2.6% (4)	03% (7)	
						
Single	0.962 (75)	0.987 (74)	0.640	97.4% (149)	97.0% (224)	2.74
Double	0.910 (71)	0.973 (73)	0.189	94.1% (144)	93.1% (215)	2.63
Triple	0.846 (66)	0.907 (68)	0.374	87.6% (134)	86.6% (200)	2.45
Quadruple	0.679 (53)	0.800 (60)	0.131	73.9% (113)	71.9% (166)	2.03
Quintuple	0.103 (8)	0.160 (12)	0.416	13.1% (20)	12.1% (28)	<1

ACR, adequate clinical response; TF, treatment failure; MOM, multiplicity of mutations; Mutation/TF ratio = prevalence of MOM (calculated)/TF rate (35.4)]. N_1_, number of genotyped samples; N_2_, total number of samples; GRI, genotype-resistance index

### Statistical analysis

The Statistical Product and Service Solutions (SPSS; version 13) software was used for the statistical analysis. Independent T-test was used to compare between subgroups of patients infected by parasites with (MOM 0 to 5) but different response (ACR and TF) regarding parasite density and patient's age. ANOVA was used for comparing parasite densities between all study groups. The gametocyte prevalence and longevity was compared between parasite infections with different number of mutations by calculating the Odds Ratio and using Chi-Square Tests, respectively.

## Results

### Clinical, parasitological and genotyping data

The clinical and parasitological data was presented before [9]. Briefly, the treatment response of the two treatment groups (SP and SP/CQ) was comparable, TF of SP alone (31.7%) and of SP plus CQ (36.6%) was similar, and the overall TF rate was 35.4% (90/254, PCR-corrected). The parasite infections used in this study were obtained from 168 patients treated with SP/CQ (n = 127; mean age 22.5 ± 16.4 Y) and SP alone (n = 41; 22.9 ± 15.5 Y). The prevalence of the individual *dhfr *mutations; 51I, 108N, 59R and 164L, were 0.89, 0.89, 0.005 and 0.0, respectively, and that of *dhps*; 437G, 540E, 581G, 436F and 613T, were; 0.88, 0.77, 0.13, 0.005 and 0.0 respectively.

### Overall prevalence of MOM, and its relation with the treatment response

**Figure 2:**
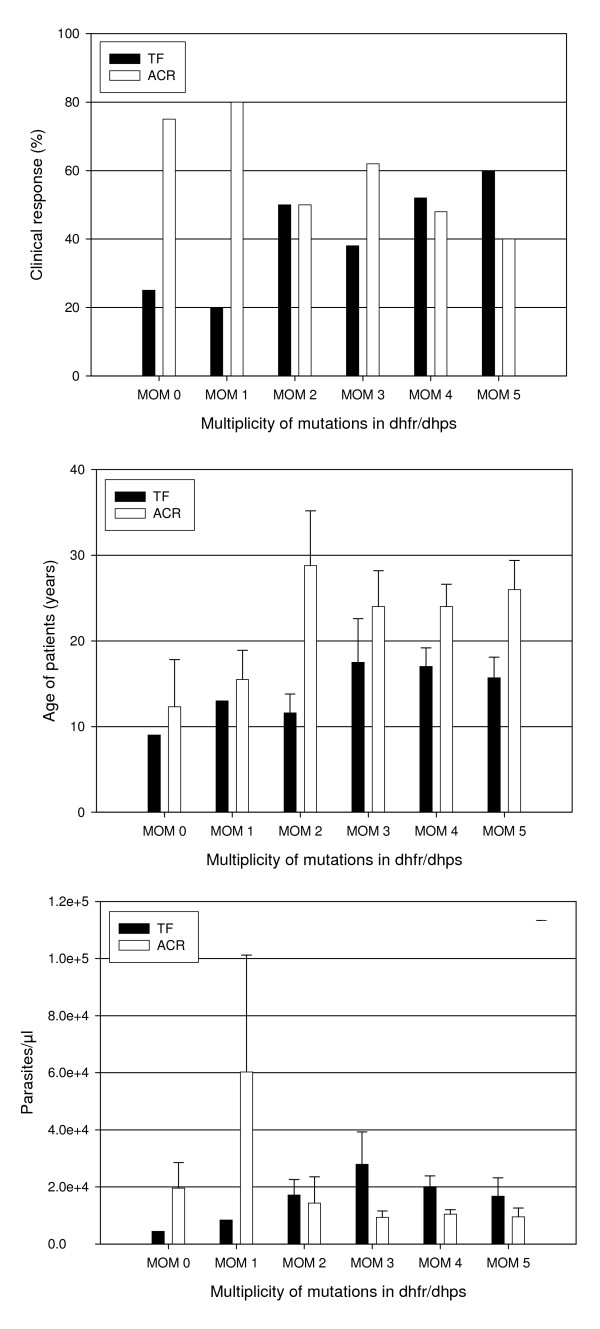
Comparisons between parasite groups of different *dhfr/dhps *genotype profile; wild parasite infections and infections with different number of mutations (MOM 1 to MOM 5), and in each parasite group between infections obtained from patients attended adequate clinical response (ACR) (white bars) and those had treatment failure (TF) (back bars). The test parameters were; A. The proportions of patients with different treatment outcome in each group (upper) B. The mean ages of the parasite donors in years (middle). C. The pre-treatment parasite density (lower). The pattern in wild infections and infections with single mutation (MOM 1) was different from that in infections with MOM 2 to 5.

The subset of analysed samples (n, 153) represented most of the SP-resistant parasite and 45% of the SP-sensitive parasite population. Still, the MOM prevalence according to the analysed samples was similar to that calculated from the total parasite population (n, 231; the remaining 23 infections were the calculated to be negative in at least one of the examined loci, if all 254 infections were genotyped) (Table 1). The overall prevalence of infections with at least one, two, three, four or five mutations (cumulative MOM 1–5), as estimated from the total population, were: 0.97, 0.93, 0.87, 0.72 and 0.12, respectively. The prevalence of cumulative MOM 0–5 was comparable between the treatment outcome groups, the ACR and TF. Only four parasite infections (0.026) were found carrying wild-type alleles in all examined loci (Table 1). Alternatively, taking all patients infected with parasites of the same MOM grade together, the proportion of patients had TF was 25% and 20% in groups of patients infected with parasites had MOM 0 and 1, respectively, and 60% in patients had infections with MOM5. The proportions of the TF in infections of MOM 2–4 ranged between the above mentioned values (Figure 2A), however, all the differences were not significant. The frequency of the MOM 0–5 (non-cumulative) was also not different between the parasite infections obtained from patients with ACR and TF response, and between ETF and LTF (data not shown). The infections with single mutation (MOM 1) were few (n = 5) and comparable to the wild-type infections (MOM 0) in number (n = 4) and other characteristics as will be shown

### Multiplicity of mutations, age and immunity

Patients with a TF outcome were significantly younger in age than patients achieving an ACR (P > 0.001), but no significant difference in age between ETF and LTF (P = 0.193). Patients infected with parasites with different number of mutations including the wild-type parasites (MOM 0–5), were not different in age (P = 0.839, ANOVA) (Figure 2B, comparison between all double bars). However, for patients infected with parasites of the same MOM grade, patients achieved ACR were mostly significantly older in age than patients had TF (Figure 2B, comparison between black and white bars in each pair separately); MOM2: (P = 0.052), MOM3: (P = 0.34), MOM4: (0.042) and MOM5: (P = 0.019). But no significant difference between the two groups (ACR *vs *TF) when infections caused by wild parasites (P = 0.79) or parasites with a single mutation (P = 0.76).

The role of immunity in clearance of mutant parasites was estimated by calculation of the ratio of cumulative MOM prevalence to the corresponding TF rate, GRI, which varied between 2.03 and 2.74 (excluding MOM 5, prevalence less than TF rate), as shown in Table 1.

### Multiplicity of mutations and pre-treatment (D0) parasitaemia

There was no significant difference in the pretreatment (D0) parasite density between ACR and TF donors of these samples (P = 0.143). However, there was a certain trend of differences in parasite density between patients infected with parasites with different *dhfr/dhps *mutations (MOM 0 to 5; P = 0.066, ANOVA). For infections of the same number of mutations (MOM), the parasite count was higher in patients with ACR outcome if the MOM grade was 0 or 1, and the reverse was true (i.e. higher count in TF) if the MOM grade was 2 to 5. However, the difference was only significant for MOM4 parasite infections, P = 0.022, Independent T-Test, (Figure 2C).

### Multiplicity of mutations and gametocytogenesis

Gametogenesis, was defined as the ability of the infections to produce gametocytes at least once during follow up (D0, 3, 7, 14, 21 and 28), while gametocyte longevity, refers to the number of times gametocytes were detected during the follow up. The later was scaled from 0 (no gametocyte detected) to 6 (gametocytes detected in the 6 days of follow up). Both parameters are indices for gametocytogenesis *in-vivo*. None of the wild-type or MOM1 infections (n = 9) produced gametocytes during the follow up, while parasites with multiple mutations (MOM 2–5) had high rates of gametogenesis (60 – 70%), the difference was strongly significant (P = 0.004, Chi-Square Tests). The odd ratios confirmed the difference in gametogenesis between MOM 0 – 1 and MOM 2 – 5 parasite infections (Table 2). For the comparison of the gametocyte longevity in infections with parasites of different MOM grades, there was significant difference between infections with MOM 0 – 1 and MOM 2 – 5, (P = 0.002), Table 2.

**Table 2:** The gametocyte rate – gametogenesis – and gametocyte longevity (gametocytogenesis) in malaria infections caused by wild-type parasites and parasites with different number of *dhfr/dhps *mutations (MOM 1 – 5). The differences in gametogenesis between parasites with different MOM were estimated by calculation of the Odd ratios with reference to MOM5.

Gametocytogenesis												
	Gametogenesis					Gametocyte longevity						
	Yes	No	Odd Ratio	95% CI								
		
				Lower	Upper	0	1	2	3	4	5	6 (max)
*Wild (4)	0	1.0	3.330	1.707	6.511	1.0	0	0	0	0	0	0
*Single (5)	0	1.0	3.330	1.707	6.511	1.0	0	0	0	0	0	0
Double (10)	0.60	0.40	1.556	0.319	7.597.	0.40	0.20	0.30	0.10	0	0	0
Triple (21)	0.62	0.38	1.436	0.391	5.269	0.38	0.33	0.19	0.05	0	0.05	0
Quadruple (93)	0.69	0.31	1.057	0.364	3.028	0.31	0.24	0.23	0.17	0.05	0	0
Quintuple (20)	0.70	0.30	1.000	0	0	0.30	0.20	0.30	0.15	0.05	0	0

* In the two groups the test parameters were significantly lower than in the other groups

## Discussion

The mutations of genes encoding important enzymes such as DHFR and DHPS, should affects parasite metabolism and indeed change certain parasite characteristics. The goal of this study is to understand the implications of gene mutations of the two enzymes in certain parasite characteristics, namely the susceptibility of the parasite to immune clearance (parasite fitness), sexual reproduction (gametocytogenesis) and asexual reproduction (parasite growth).

The *dhfr/dhps *gene mutations are known to be involved in parasite resistance to SP [2,3], and *in-vivo *studies have shown that immunity is involved in clearance of drug resistant parasites [9,13]. Taken together, the *dhfr/dhps *mutations are probably involved in immune clearance of SP resistant infections. In this study the ratio of TF to prevalence of the predominant quadruple *dhfr/dhps *mutations (MOM 4) was approximately 1: 2, that was a circumstantial evidence for the role of host immunity in clearance of mutant parasites. The supporting evidence was the higher age (a surrogate marker for immunity) of the successfully treated patients. Interestingly, the ratio of MOMs prevalence to the actual treatment failure (2.03 and 2.74) is similar to the GRI (genotype resistance index) in Mali (1.6 to 2.8), a region epidemiologically similar to this study site [14], although different drugs were used. The specific evidence for the increased suscebtibilty of the *dhfr/dhps *multi-mutant parasites to immune clearance was the difference in age of the patients infected with parasites with the same number of mutations (MOM grade) but had different treatment outcome. This difference in age (ACR vs TF) was limited to the parasite infections with multiple mutations (MOM 2–5), but not to infections with wild parasites and parasites with single mutation (Figure 2B). For identification of the most important mutations (loci), larger sample size is needed, however, in this study, 51I, 108N, 437G and 540E were the predominant mutations (Figure 1). Furthermore, the reduced resistance of multi-mutant parasites to host immunity is a sign of reduced fitness, supporting previous reports [4,5,15]. The degree of immunity which is required for clearance of parasite with specific number of mutation was estimated by the GRI value. The interpretation of this data indicates that, there are at least three factors directly contributing to parasite clearance (ACR): the drug (SP), immunity (represented by age) and parasite mutations (MOM > 1). The paradox is that, the *dhfr/dhps *mutations provide the parasite the ability to resist chemotherapy, while it renders it more susceptible to the host immunity.

The pre-treatment parasite density probably reflects the *in-vivo *parasite growth, assuming that sequestration is confined to severe malaria infection; and the peripheral parasitaemia represents the total parasite load. In this study, the parasite growth was influenced by *dhfr/dhps *mutations where growth of wild and parasites with single mutation (MOM 1) was relatively higher from that of multi-mutant parasites in patients achieved ACR and the reverse was true in case of TF (Figure 2C). However, these results (trends) need to be taken with precaution because the sample size was small and the accuracy of the microscopic estimation of parasitaemia is not high, in addition parasite sequestration is not confined to severe malaria. In a previous study in the same site it was found that, there was significant association between reduced parasite growth and *dhfr/dhps *mutations [15].

It is known that, the frequency of gametocytaemia increases with treatment failure [16], treatment with SP [17] and younger age [9]. In this study, the gametogenesis was significantly higher in parasites with multiple *dhfr/dhps *mutations compared to wild type and parasites with single mutation, as recognized in other studies [18,19]. However, only two *dhfr/dhps *mutations (MOM2) were needed to enhance the gametocytogenesis significantly, with no additive effect for more mutations (MOM 3–5). In the in-*vivo *study, the patient achieved ACR had significantly lower frequency of gametocytaemia compared to patients who had TF. The association between gametocytogenesis and *dhfr/dhps *mutations was independent of the treatment outcome and the host age. That is because, there was no association between the treatment outcome and the mutations in this study, and the age of patients infected with wild-type parasite and parasites with MOM1 (median 13 years) was not different from the age of patients who had TF (median, 12 years). Also, the differences cannot be due to gametocytogenic effect of SP treatment, as all patients were treated with SP. Although, the gametocytogenesis is the corner stone in the spread of the resistant strains, there are only few studies that had evoked the issue at the molecular level [15,19]. Finally, it is difficult to look at these parasite characteristics of gametocytogenesis, immune clearance and *in-vivo *parasite growth in isolation of drug resistance since they are not independent from each other, thus, further studies are needed for detailed molecular exploration.

In conclusion, the *dhfr/dhps *gene mutations in *P. falciparum *are probably affecting the parasite fitness by rendering the parasite more susceptible to immune clearance than the wild-type variants. The increased immune clearance of mutant parasites can explain the lack of association between the prevalence of *dhfr/dhps *mutations and the TF rate in this setting. Furthermore, there were indications that the *in-vivo *asexual growth of the *dhfr/dhps *multi-mutant parasites was lower compared to wild types, while the gametocytogenesis in the former group was significantly higher than in the later. That is more likely an evolutionary mechanism compensating for the reduced asexual reproduction, and also can explain the fast propagation of SP resistance. Finally, the above-mentioned implications need only two (double) mutations in the *dhfr/dhps *genes irrespective of their codon/gene locus, while it was reported, SP resistance is associated with higher number of mutations in specific loci.

## Competing interests

The author(s) declare that they have no competing interests.

## Authors' contributions

IAE, MIE and HAG were contributed in all aspects of the study. IFK, MA and IB, were contributed in laboratory work, interpretation of results and paper writing. EM, contribution was basically in statistical analysis and paper writing
